# Review of "*Insect Pathogens Molecular Approaches and techniques*" by S. P. Stock, J. Vandenberg, I. Glazer and N. Boemare

**DOI:** 10.1186/1756-3305-2-28

**Published:** 2009-07-01

**Authors:** Hilary Hurd

**Affiliations:** 1 School of Life Science, Keele University, Staffordshire, ST5 5BG, UK

## Review

Insects are not only of immense economic and medical importance, they provide important models for the advancement of knowledge and many are of ascetic value. They are also beset by an array of pathogens, despite their well developed defence systems. Interest in these pathogens revolves around their potential use as biological control agents for insect pests or the desire to protect beneficial insects against infection. The adoption of molecular approaches to the investigation of insect pathogens and pathogen-insect interactions has had a large impact on research and this volume provides a timely review of how these techniques are being applied.

The multi authored book, consisting of sixteen chapters, is broken down into four themes; identification and diagnostics, evolutionary relationships and population genetics, host-pathogen interactions and genomics and genetic engineering. The chapters follow a standard format and consist of several short sections covering a wide range of topics related to the chapter heading. In some cases this results in a superficial overview, but all chapters contain a comprehensive list of references so that readers can follow up areas of specific interest. Most chapters deal with particular taxa of entomopathogenic organisms thus the reader may wish to focus upon chapters pertaining to their particular study group or take a comparative approach. Emphasis throughout is on molecular techniques, often discussed and evaluated from the point of view of the authors' personal experience. Although a useful glossary is provided the extensive use of acronyms warrants, in addition, a list of abbreviations.

In the first section of the book authors of all the chapters emphasise the importance of molecular techniques to the revision of taxonomy and classification on a phylogenetic basis. Likewise in the second section authors emphasise the significant changes made to hypothesis concerning the evolutionary biology of insect pathogens following the application of DNA sequencing and analytical methods. Chapters on host-pathogen interactions cover an eclectic range of topics including the development of a baculovirus expression vector, tsetse fly immune responses and gene expression in tripartite nematode-bacterium-insect symbiosis. In the last chapter in this section and those in the final section on genomics and genetic engineering, detailed overviews of molecular strategies are given, a case study approach is frequently taken and future prospects and pitfalls discussed.

As someone not directly involved with the fields of work presented in this book I found aspects of all chapters interesting. The overviews of techniques may prove valuable to PhD students or researchers considering taking a molecular approach to their work on insect pathogens however, consultation of detailed technical manuals is likely to be required before investigations can begin.

## Competing interests

The author declares that they have no competing interests.

